# Design of a Compact Multiband Monopole Antenna with MIMO Mutual Coupling Reduction

**DOI:** 10.3390/s24175495

**Published:** 2024-08-24

**Authors:** Chang-Keng Lin, Ding-Bing Lin, Han-Chang Lin, Chang-Ching Lin

**Affiliations:** 1 Department of Electronic and Computer Engineering, National Taiwan University of Science and Technology, Taipei 10607, Taiwan; d10902007@mail.ntust.edu.tw (C.-K.L.); d10802001@mail.ntust.edu.tw (H.-C.L.); 2 Department of Electrical Engineering, Yuan Ze University, Taoyuan 320315, Taiwan; timlin650219@msn.com

**Keywords:** multiband, monopole antenna, Wi-Fi, MIMO, mutual coupling, mutual coupling suppression, IoT

## Abstract

In this article, the authors present the design of a compact multiband monopole antenna measuring 30 × 10 × 1.6 mm^3^, which is aimed at optimizing performance across various communication bands, with a particular focus on Wi-Fi and sub-6G bands. These bands include the 2.4 GHz band, the 3.5 GHz band, and the 5–6 GHz band, ensuring versatility in practical applications. Another important point is that this paper demonstrates effective methods for reducing mutual coupling through two meander slits on the common ground, resembling a defected ground structure (DGS) between two antenna elements. This approach achieves mutual coupling suppression from −6.5 dB and −9 dB to −26 dB and −13 dB at 2.46 GHz and 3.47 GHz, respectively. Simulated and measured results are in good agreement, demonstrating significant improvements in isolation and overall multiple-input multiple-output (MIMO) antenna system performance. This research proposes a compact multiband monopole antenna and demonstrates a method to suppress coupling in multiband antennas, making them suitable for internet of things (IoT) sensor devices and Wi-Fi infrastructure systems.

## 1. Introduction

In today’s digital age, the Internet has become an integral part of our daily lives. From smartphones and laptops to smart homes and IoT devices, people’s demand for faster, more reliable wireless connections continues to grow. These demands bring significant challenges to wireless communication technology, including the need for larger bandwidth and more antennas. Therefore, the Wi-Fi Alliance proposed the latest Wi-Fi 7 standard to address these demands. The Wi-Fi 7 standard features tri-band operation (supporting 2.4 GHz, 5 GHz, and 6 GHz bands), ultra-wide 320 MHz channels, 4096 QAM modulation, up to 16 × 16 MIMO, and multi-link operation (MLO) [1]. 3GPP also launched the fifth generation of mobile communications (5G). The performance goals of 5G include high data rates, reduced latency, energy savings, cost reduction, increased system capacity, and large-scale device connectivity. 5G NR (New Radio) includes the FR1 (sub-6 GHz) and FR2 (mmWave) frequency ranges [2,3]. FR1 covers frequencies from 410 MHz to 7.125 GHz, with 3.5 GHz being the most popular. FR2 covers frequencies from 24.25 GHz to 52.6 GHz. 

Therefore, many researchers have proposed UWB antennas [4,5] to satisfy the required frequency bands. However, these antennas often have larger dimensions and do not cover the Wi-Fi 2.45 GHz band. Many multiband antennas [6,7,8,9,10,11,12,13] have been proposed, but they also do not cover the required frequency bands. A method using loading metamaterials to achieve multifrequency operation in monopole antennas is proposed in [14]. Another more complex method, using two-dimensional (2-D) resonant-type composite right/left-handed transmission lines to achieve multiband and high gain in patch antennas, is proposed in [15]. This method can cascade to three or five cells as a series patch antenna with beam steering functionality. In multiple antenna MIMO systems, a critical issue is the reduction of mutual coupling between antennas, especially at lower frequencies. In [6], a miniaturized antenna with engraved inductor and capacitor is presented. This design utilizes opposite conduction currents on the two radiators to eliminate coupling. In [7], mutual coupling reduction is achieved by using different polarization directions and a cross-shaped ground resonator. In [8,12], a similar ground resonator is employed to achieve mutual coupling reduction. In [13], neutralization lines are connected between two antennas to achieve mutual coupling reduction and improve the reflection coefficient. In [16,17,18], four different polarization directions are used to suppress mutual coupling. However, this method is more challenging to implement in real applications. In [19], a metamaterial PCB with split-ring resonators is added between two dielectric resonator antennas to suppress mutual coupling. The split-ring resonators can be designed for the required suppression frequency. However, adding the metamaterial PCB will increase the overall size of the antenna. In [20,21], similar single-negative electric metamaterials (MTMs) were placed in the middle between two closely spaced ($\lambda_{o}/8$) patch antennas to achieve excellent mutual coupling reduction.

In this paper, the authors propose a method to suppress mutual coupling between antennas, which can be designed for the required suppression frequencies without the need for additional components or an increase in the overall size of the antenna.

## 2. Design of Antenna Structure

In this chapter, the authors introduce the compact multiband monopole antenna structure and its MIMO mutual coupling reduction method in detail, as well as their simulation results obtained using the full-wave simulation tool Ansys HFSS. The antennas’ simulation conditions are printed on a single-layer FR4 board with a relative permittivity ($\varepsilon_{r}$) of 4.4, a tangent loss (δ) of 0.02, a substrate thickness of 1.6 mm, and a copper thickness of 35 um. 

### 2.1. The Compact Multiband Monopole Antenna

**Figure 1:**
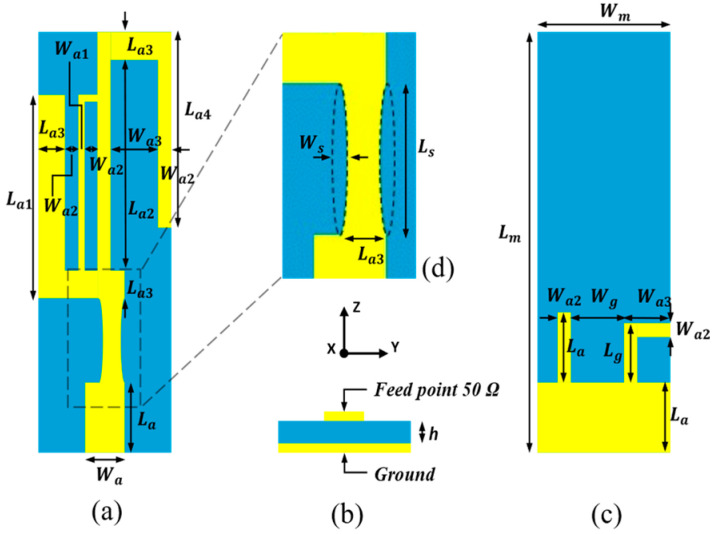
The structure of the compact multiband monopole antenna. (**a**) Front view. (**b**) Bottom view. (**c**) Back view. (**d**) Detail view.

**Figure 2:**
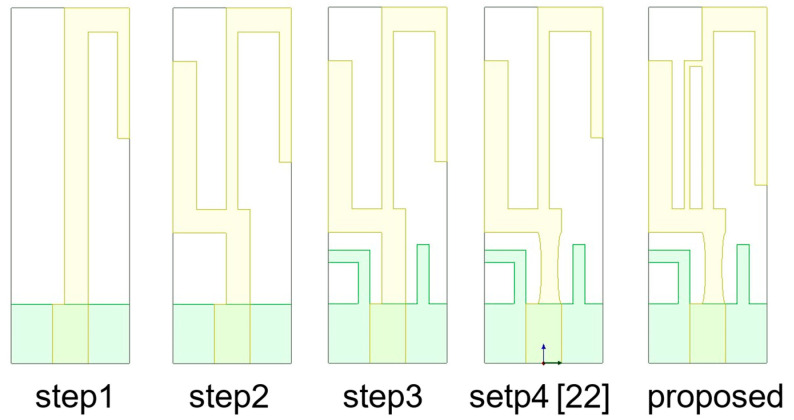
The evolutionary steps of the compact multiband monopole antenna structure.

**Figure 3:**
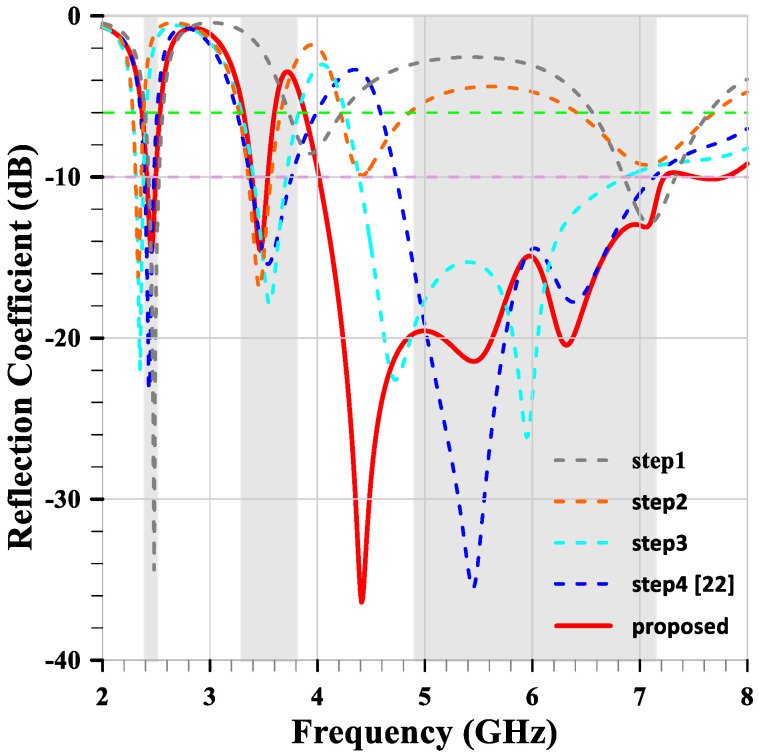
The evolution reflection coefficients of the five-step antennas. The gray background bars represent the designed target bands.

The proposed compact multiband monopole antenna in this paper evolves from the design presented in [22]. The structure and detailed dimensions of the compact multiband monopole antenna are shown in Figure 1 and Table 1. Figure 2 shows the evolutionary steps of the compact multiband monopole antenna structure. Step 1 is a quarter-wavelength monopole antenna for 2.45 GHz, calculated using Equation (1). In step 2, an arm is added to the left side for 3.5 GHz. In step 3, two arms are added to the ground at the bottom layer for 5–6 GHz. In Step 4, two elliptical cuts remove a bit of metal from the middle arm for better impedance matching, as shown in Figure 1d and published in [22]. In the proposed design, an arm (which can be seen as a piece of inductance) parallel to the middle arm creates more bandwidth from 4 GHz to 7.2 GHz compared to [22], which creates about 800 MHz bandwidth at the 4 GHz band, as shown in Figure 2 and Figure 3.

Here, $L_{arm}$ represents the length of the arms, $C_{0}$ represents the speed of light, $f_{0}$ is the designed frequency resonance, and $\varepsilon_{eff}$ represents the effective permittivity.

(1) $$L_{arm}=\frac{C_{0}}{4f_{0}\sqrt{\varepsilon_{eff}}}$$

Figure 3 shows the reflection coefficients of the five-step antennas. The under −10 dB bandwidth becomes progressively wider with each evolution step, and the impedance matching improves, especially at higher bands. The reflection coefficient of the proposed antenna has six frequency resonant points: 2.46 GHz, 3.47 GHz, 4.41 GHz, 5.45 GHz, 6.32 GHz, and 7.06 GHz, respectively. These frequency resonance points determine and satisfy the bandwidth and performance requirements for the compact multiband monopole antenna.

**Figure 4:**
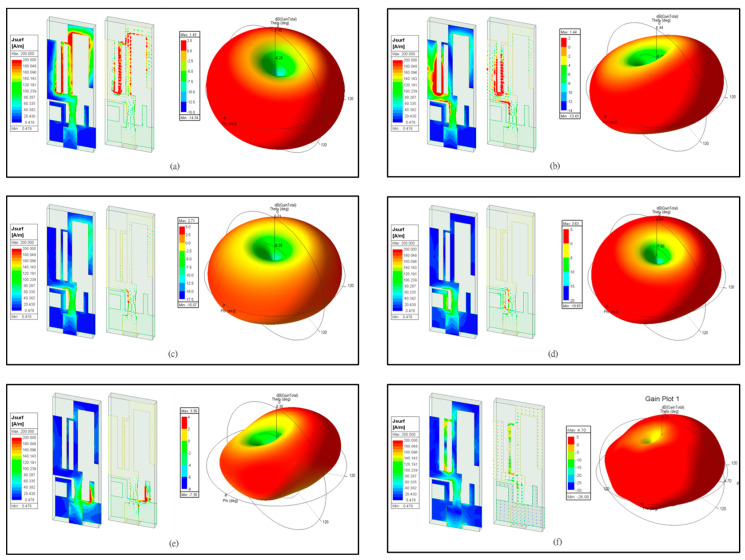
The current distributions and radiation patterns of the compact multiband monopole antenna. (**a**) 2.46 GHz. (**b**) 3.47 GHz. (**c**) 4.41 GHz. (**d**) 5.45 GHz. (**e**) 6.32 GHz. (**f**) 7.06 GHz.

Figure 4 illustrates the simulation current distributions and radiation patterns of the compact multiband monopole antenna at 2.46 GHz, 3.47 GHz, 4.41 GHz, 5.45 GHz, 6.32 GHz, and 7.06 GHz, respectively. These radiation patterns exhibit omnidirectional characteristics with gains of 1.43 dBi, 1.44 dBi, 2.71 dBi, 3.63 dBi, 3.35 dBi, and 4.7 dBi, respectively.

Figure 4a shows the current distribution of the antenna at the resonance frequency of 2.46 GHz, which exhibits the standard quarter-wavelength current distribution along the middle arm. This means the maximum current occurs at the shorted end and decreases to zero at the open end. Figure 4b exhibits a U-shaped and half-wavelength current distribution along the left arm and the parallel arm at 3.47 GHz. This means the maximum current occurs in the middle of the U-shape and decreases to zero at the two open ends. Despite the opposite current directions, they do not cancel each other out and still radiate electromagnetic power effectively. Figure 4c shows the three-quarters wavelength current distribution along the middle arm at 4.41 GHz. This means the current flows from maximum to zero, then to another maximum, and back to zero, with the current direction remaining mostly in phase. This frequency resonance point is key to creating more bandwidth. Figure 4d exhibits another U-shaped and half-wavelength current distribution along the left arm of the ground and the middle arm at 5.45 GHz. The current flows similarly to Figure 4b and still radiates electromagnetic power effectively. Figure 4e exhibits a current distribution similar to Figure 4d but with the distribution along the right arm of the ground at 6.32 GHz, where the current flows similarly to Figure 4b and radiates electromagnetic power effectively. Figure 4f shows a different current distribution. The current along the half-wavelength rectangular slot radiates electromagnetic power. This rectangular slot is formed by the middle arm and the parallel arm. The current flows similarly to an inductive slot resonator, with zero current occurring in the middle of the longer side of the rectangular slot and maximum current appearing at the shorter sides of the rectangular slot. 

**Figure 5:**
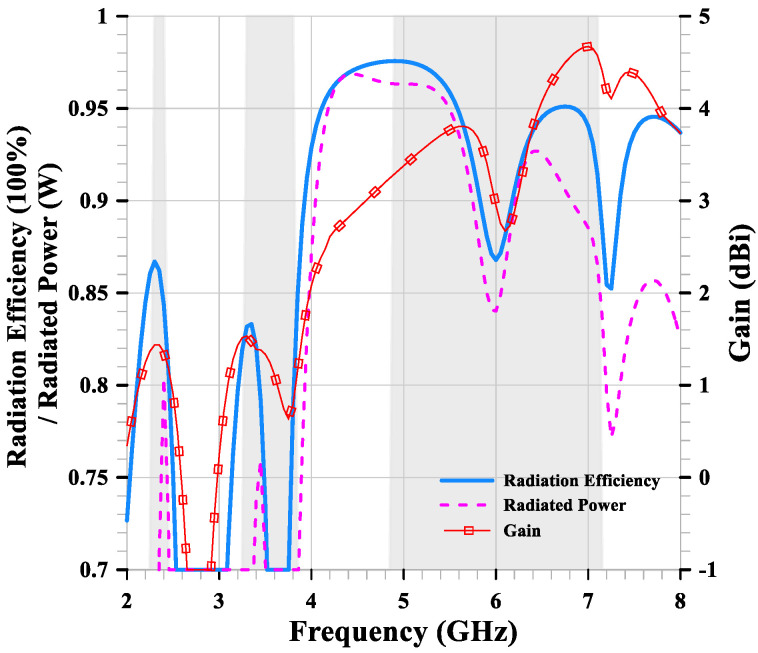
The radiation efficiency, radiated power, and gain of the compact multiband monopole antenna.

Figure 5 shows that the simulation of radiation efficiency is mostly above 70% within the required bands, with peak values of 86.7%, 83.3%, 97.5%, and 95.1% occurring at 2.3 GHz, 3.3 GHz, 5 GHz, and 6.7 GHz, respectively. The simulated radiated power mostly matches the radiation efficiency, with peak values of 0.8 W, 0.75 W, 0.97 W, and 0.93 W occurring at 2.4 GHz, 3.45 GHz, 4.4 GHz, and 6.4 GHz, respectively. The gain is consistently positive within the required bands, with peak values of 1.43 dBi, 1.44 dBi, 3.8 dBi, and 4.7 dBi occurring at 2.46 GHz, 3.47 GHz, 5.7 GHz, and 7 GHz, respectively. Due to the superposition of currents, the radiation pattern exhibits higher gain at higher frequencies, but it also creates an unbalanced omnidirectional radiation pattern. 

### 2.2. MIMO Antenna Mutual Coupling Reduction

In this section, the authors introduce an effective method to suppress MIMO mutual coupling. The design targets for suppression are frequencies at 2.46 GHz and 3.47 GHz because lower frequencies easily produce mutual coupling interference, causing data errors in the MIMO antenna. The material of the MIMO antenna is the same as that of the single antenna, FR4, with the same characteristic parameters. The coordinates are also the same as in Figure 1.

**Figure 6:**
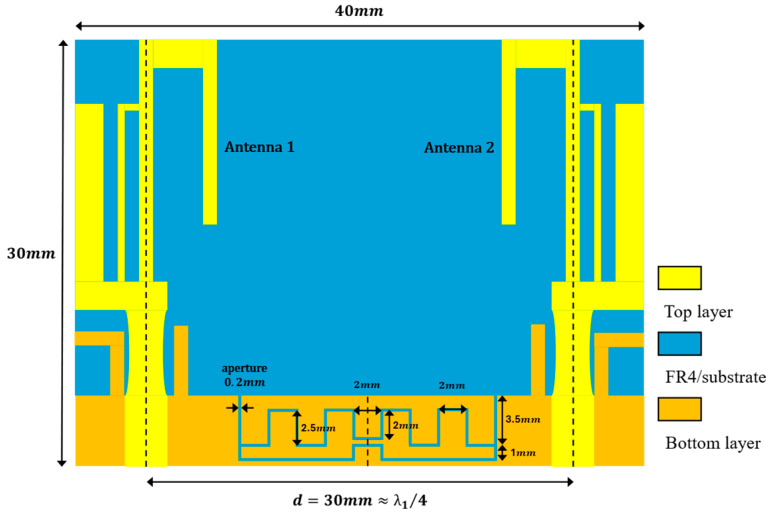
The configuration and dimensions of the MIMO antenna structure, including two meander slits.

The configuration and dimensions of the MIMO antenna structure are shown in Figure 6. The distance between antenna 1 and antenna 2 is 30 mm, which corresponds to a quarter-wavelength of the lowest resonance frequency 2.46 GHz. The 30 mm distance is considered for a scenario where WiFi-7 standard access points (APs) need to place 16 antennas [1] within a limited space. However, because the two antennas are close to each other, the lower two frequencies experience strong mutual coupling. Therefore, the design targets for suppression are these frequencies. The two antennas are placed opposite each other and share a common ground. The middle of the ground has two meander slits, with the slits having a common open end on both sides. The length of the upper meander slit is 41 mm, and the lower meander slit is 29 mm, corresponding to half-wavelengths at 2.46 GHz and 3.47 GHz, respectively. Here, the length of the slits is calculated using the same method as Equation (1), with the only change being the constant coefficient adjusted to 2. The effective permittivity derivative is calculated using Equation (2) and set to approximately 2.2. Here, $\varepsilon_{r}$ represents relative permittivity.

(2) $$\varepsilon_{eff}=\sqrt{\varepsilon_{r}+1}$$

**Figure 7:**
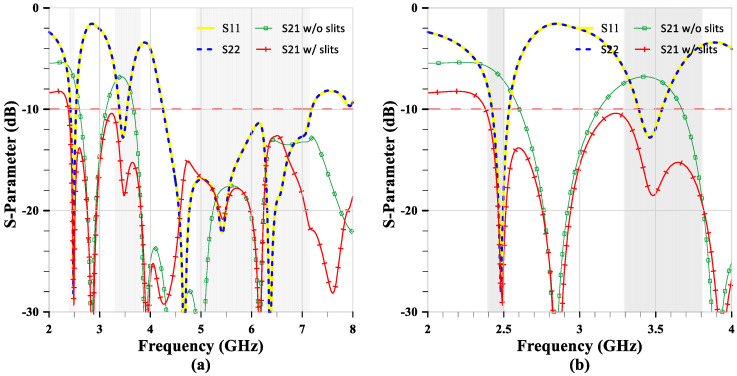
The S-parameters of the MIMO antenna. (**a**) The full band. (**b**) The partial band.

Figure 7a shows the simulation S-parameters of the MIMO antenna. The reflection coefficients $S_{11}$ and $S_{22}$ are the same, and their bandwidths are similar to the single element, with the only difference starting at the higher band from 4.2 GHz. Figure 7b exhibits the detailed transmission coefficient or isolation $S_{21}$, with and without slits on the ground. Comparing the $S_{21}$ with and without slits shows mutual coupling suppression from −6.5 dB and −6.8 dB to −29.4 dB and −18.5 dB at 2.46 GHz and 3.47 GHz, respectively. The suppression is 22.9 dB and 11.7 dB, corresponding to a reduction in mutual coupling by 195 and 14.8 times. In the higher frequency band, the mutual coupling is always under −15 dB, indicating sufficient distance between elements. However, there is a peak at −12 dB at 6.5 GHz because the effective wavelength ($\lambda_{e}$) is 19.86 mm at 6.5 GHz ($\lambda_{e}=\frac{C_{o}}{f_{o}\sqrt{\varepsilon_{e}}}$). The 30 mm antenna separation distance is approximately 1.5$\lambda_{e}$, which excites resonance and causes stronger mutual coupling, resulting in peak isolation at −12 dB.

**Figure 8:**
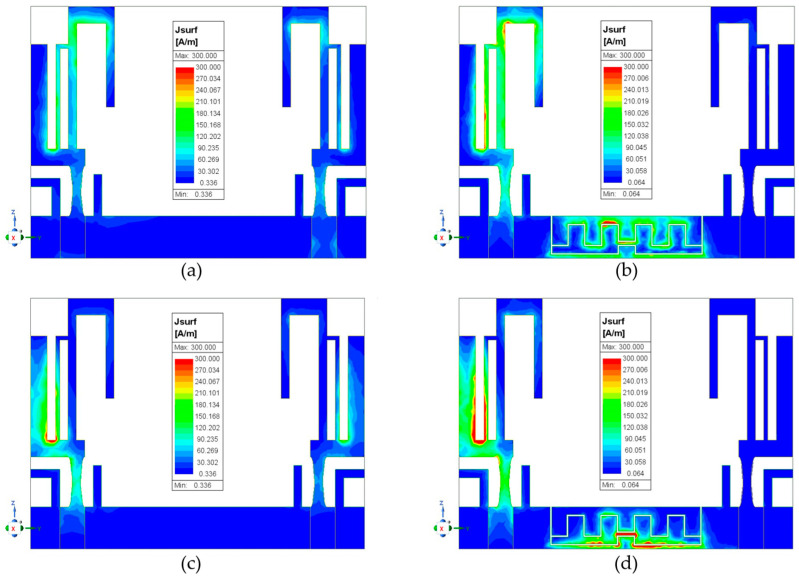
The current distributions of the MIMO antenna with antenna 1 excited and antenna 2 terminated. At 2.46 GHz without slits (**a**) and with slits (**b**). At 3.47 GHz without slits (**c**) and with slits (**d**).

A more direct way to observe the effect of the method for reducing mutual coupling is from the current distribution of the MIMO antenna. Figure 8 shows the current distribution of the MIMO antenna. Observing Figure 8a, one can see that antenna 1’s electromagnetic interference causes an inductive current on antenna 2 when antenna 1 is excited at 2.46 GHz and antenna 2 is terminated. However, in Figure 8b, there is no inductive current on antenna 2; instead, the conductive current is observed on the upper meander slit. Therefore, the authors believe that the mutual coupling path consists of two components: one from the electromagnetic (EM) wave and the other from the common ground. Most of the coupling energy likely follows the second path, as Figure 8b demonstrates that the coupling energy is absorbed or re-radiated into the air by the upper meander slit on the ground. From the observed conductive current on the upper meander slit, which has maximum current in the middle and zero current at both open ends, it is evident that this condition matches the previously calculated half-wavelength slit. Furthermore, in Figure 8c,d, the conditions are similar to the previous observations, with only the excitation frequency changing to 3.47 GHz and the decoupled conductive current observed on the lower meander slit. 

**Figure 9:**
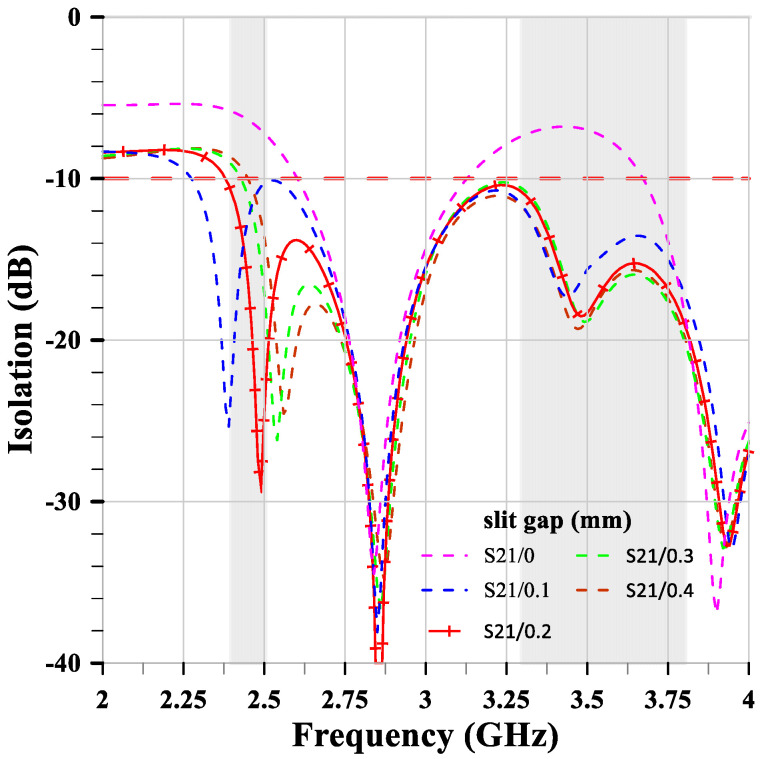
The relationship between isolation and the slit gap.

The relationship between isolation and slit gap is shown in Figure 9. The gap of the two slits is the same size. When the gap changes from 0.1 mm to 0.4 mm (with 0 mm representing no slits), the isolation remains largely unchanged, with suppression around −18 dB at 3.47 GHz. However, there is a significant change at 2.46 GHz. As the gap increases, the resonant frequency shifts from lower to higher values. Therefore, the authors chose a gap of 0.2 mm for the manufacturing parameter because it provides the correct resonant frequency at 2.46 GHz and achieves deeper suppression at −29.4 dB.

**Figure 10:**
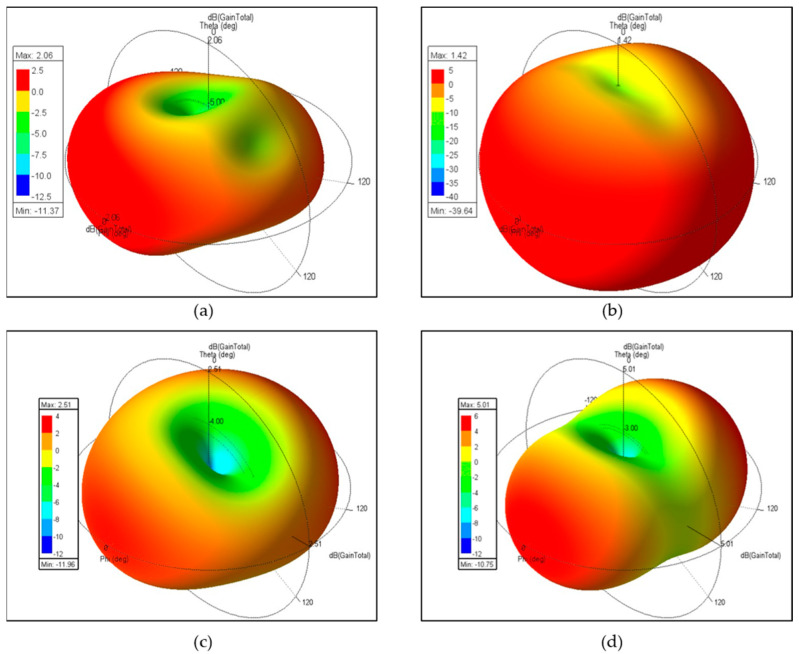
The radiation pattern of the MIMO antenna at 2.46 GHz: (**a**) without slits and with antenna 1 excited and antenna 2 terminated, or (**b**) with both antennas excited; (**c**) with slits and with antenna 1 excited and antenna 2 terminated, or (**d**) with both antennas excited.

Figure 10 shows the radiation patterns of the MIMO antenna at 2.46 GHz. There are no slits on the common ground. When antenna 1 is excited and antenna 2 is terminated, as shown in Figure 10a, and when both antennas are excited, as shown in Figure 10b, different radiation patterns are exhibited, with gains of 2.06 dBi and 1.42 dBi, respectively. Due to the absence of slits, there is no way to suppress mutual coupling, so antenna 1 and antenna 2 interfere with each other, causing a decrease in gain. From an antenna array perspective, theoretically doubling the elements should increase the gain by 3 dB. Therefore, there is no constructive interference, only destructive interference. However, Figure 10c,d present a different situation with slits on the common ground. When antenna 1 is excited and antenna 2 is terminated, the gain is 2.51 dBi, higher than the gain shown in Figure 10a. This leads the authors to believe that the conductive current on the upper meander slit is not absorbed by the ground but, instead, radiates into the air. Furthermore, observing Figure 10d, the gain is 5.01 dBi, not 5.51 dBi (which would be the expected sum of 2.51 dBi + 3 dBi). When both antenna 1 and antenna 2 are excited in phase, their conductive currents on the upper meander slit are opposite and thus cancel each other out, as shown in Figure 12a. This results in no radiation from the slit, and the gain observed is due only to the constructive interference between antenna 1 and antenna 2, creating a more directional radiation pattern. 

**Figure 11:**
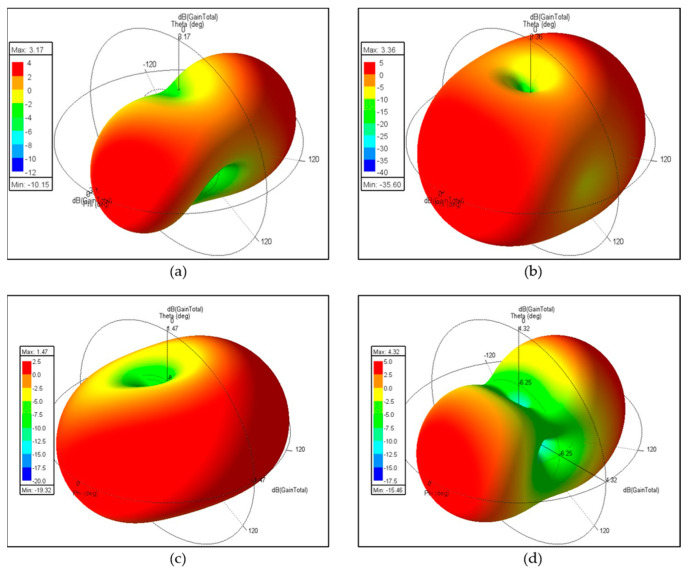
The radiation pattern of the MIMO antenna at 3.47 GHz: (**a**) without slits and with antenna 1 excited and antenna 2 terminated, or (**b**) with both antennas excited; (**c**) with slits and with antenna 1 excited and antenna 2 terminated, or (**d**) with both antennas excited.

**Figure 12:**
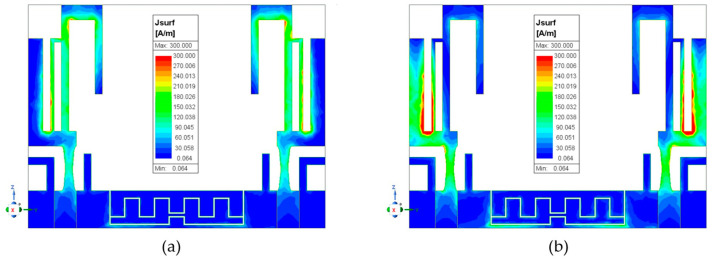
The current distributions of the MIMO antenna with both antenna 1 and antenna 2 excited. (**a**) At 2.46 GHz with slits. (**b**) At 3.47 GHz with slits.

Figure 11 shows the radiation patterns of the MIMO antenna at 3.47 GHz. These situations are similar to those observed at 2.46 GHz. However, without slits on the common ground, the increased distance between the radiators at 3.47 GHz results in less interference, leading to no decrease in gain but rather a slight increase from 3.17 dBi to 3.36 dBi, as shown in Figure 11a,b. Figure 11b also exhibits a directional radiation pattern due to the increased distance between the two radiators, which results from the shorter wavelength. Furthermore, with slits on the common ground, a comparison of Figure 11c,d shows pattern gains of 1.47 dBi and 4.32 dBi, respectively. The increase in gain is 2.85 dB, which is quite close to the theoretical value. Higher frequencies and shorter wavelengths indeed benefit the radiation pattern. The conductive currents cancel each other out, as shown in Figure 12b.

## 3. Antenna Implementation and Measurement Results

**Figure 13:**
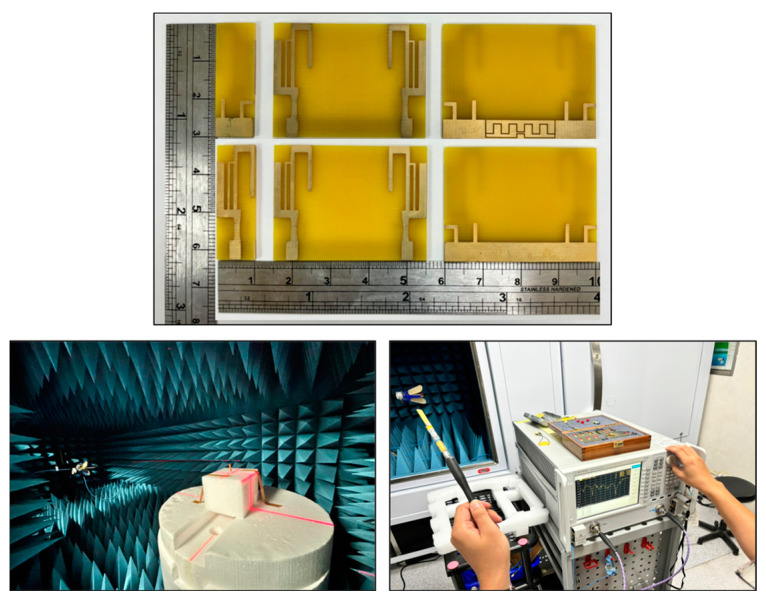
The prototype antennas, the network analyzer, and the measurement environment.

This chapter covers the implementation details and the measurement results of the proposed antenna designs, as shown in Figure 13. The antennas were fabricated on a single-layer FR4 board with a thickness of 1.6 mm, a relative permittivity ($\varepsilon_{r}$) of 4.4, and a copper thickness of 35 um. Their performance was evaluated using an Agilent PNA network analyzer—N5277A and an anechoic chamber. The key parameters measured include reflection coefficients, radiation patterns, and gain.

### 3.1. The Compact Multiband Monopole Antenna

**Figure 14:**
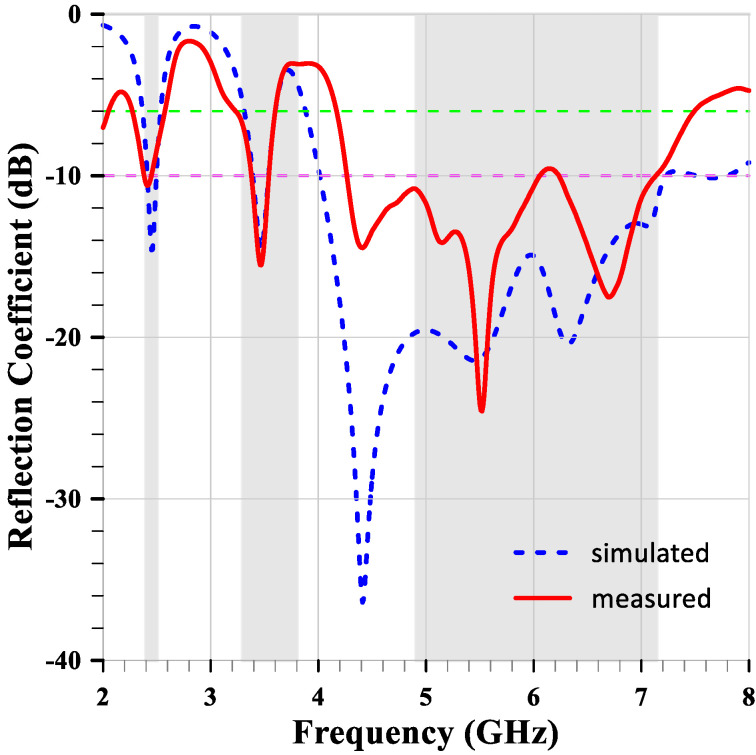
The measured and simulated reflection coefficients of the compact multiband monopole antenna.

Figure 14 shows the measured and simulated reflection coefficients of the compact multiband monopole antenna. The measurements indicate good agreement with the simulations, validating the design of the antenna. The antenna exhibits multiband characteristics with reflection coefficients below −10 dB at the targeted frequency bands. However, at 2.46 GHz, the reflection coefficient is only −10.2 dB, and it is almost the same at 3.47 GHz. Additionally, within the highest band, from 4.4 GHz to 7.1 GHz, the measured bandwidth is 400 MHz less than the simulated bandwidth. 

**Figure 15:**
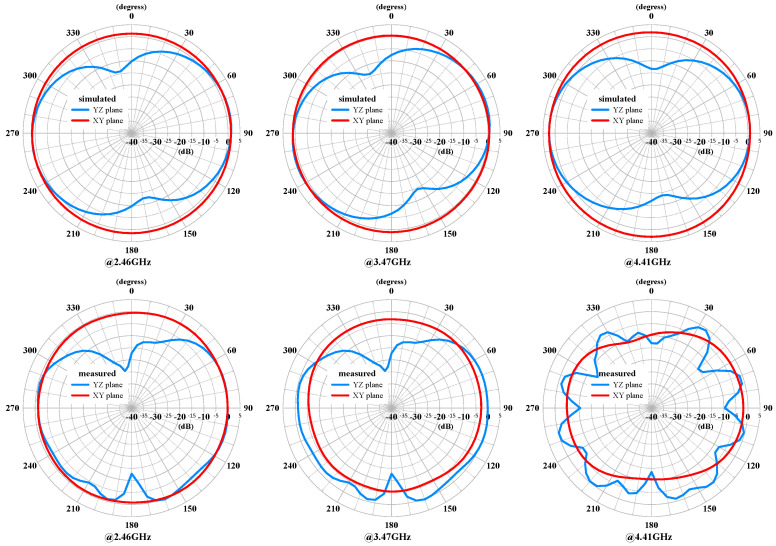
The measured and simulated radiation patterns of the compact multiband monopole antenna at 2.46 GHz, 3.47 GHz, and 4.41 GHz.

**Figure 16:**
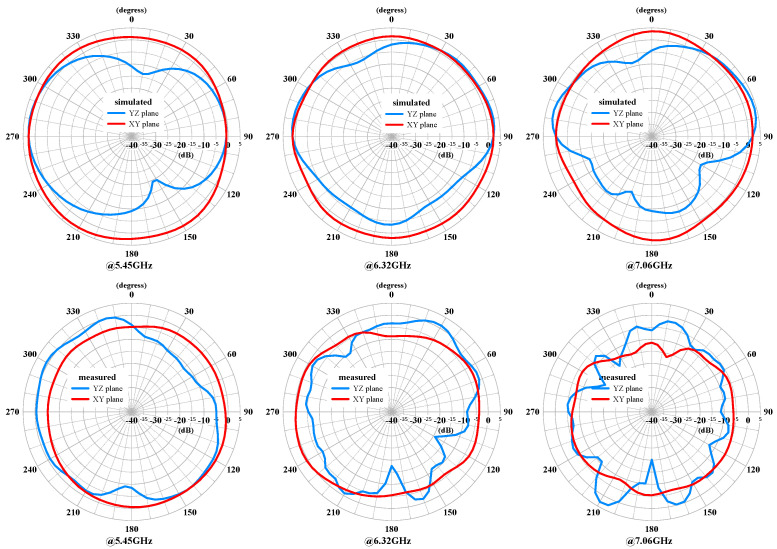
The measured and simulated radiation patterns of the compact multiband monopole antenna at 5.45 GHz, 6.32 GHz, and 7.02GHz.

The measured and simulated radiation patterns of the compact multiband monopole antenna are shown in Figure 15 and Figure 16, respectively. The patterns demonstrate good omnidirectional coverage in the azimuth angle (XY plane) and stable performance across the different frequency bands. However, the gain decreases noticeably as the frequency increases. The elevation angle (YZ plane) also exhibits a similar trend and is not smooth. The gain decrease is likely due to the loss from the FR4 PCB and the SMA connector as the frequency increases.

### 3.2. MIMO Mutual Coupling Reduction

**Figure 17:**
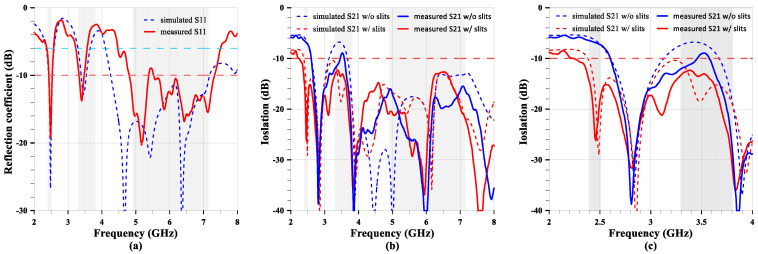
The measured and simulated S-parameters of the MIMO antenna. (**a**) The reflection coefficient. (**b**) The isolation of the full band. (**c**) The isolation of the partial band.

The measured and simulated reflection coefficients of the MIMO antenna with slits, with both antennas excited, are shown in Figure 17. The measurements indicate good agreement with the simulations at the lower two bands, but there is a narrower bandwidth, as shown in Figure 17a, at the higher band. However, the reflection coefficient is almost below −10 dB at that band and covers the required bandwidth. The measured and simulated isolations of the MIMO antenna with and without slits, with both antennas excited, are shown in Figure 17b,c. Those isolations follow a similar trend and are below −13 dB at the higher band. Furthermore, Figure 17c shows the difference between the MIMO antennas with and without slits. The simulated and measured curves are quite close at the 2.4 GHz band, highlighting an impressive reduction in mutual coupling. However, there is only a 4 dB reduction in mutual coupling at 3.47 GHz, but another suppression response occurs at 3.2 GHz, showing the same level of isolation as the simulation. This may be the designed suppression frequency, but the lower meander slit is too long for some unknown reason. Additionally, there are two response frequencies at 2.8 GHz and 3.9 GHz, respectively. These frequencies result from the physical size and first-order harmonics, and all four curves exhibit the same response frequencies.

**Figure 18:**
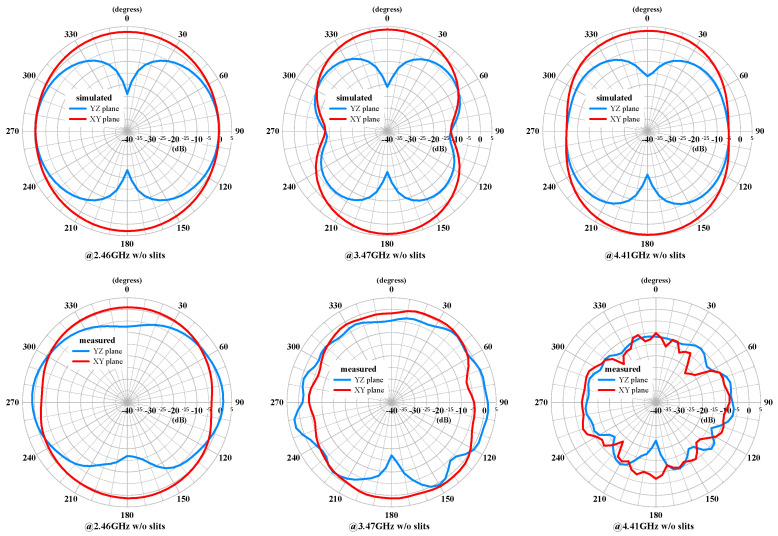
The measured and simulated radiation patterns of the MIMO antenna with slits at 2.46 GHz, 3.47 GHz, and 4.41 GHz.

**Figure 19:**
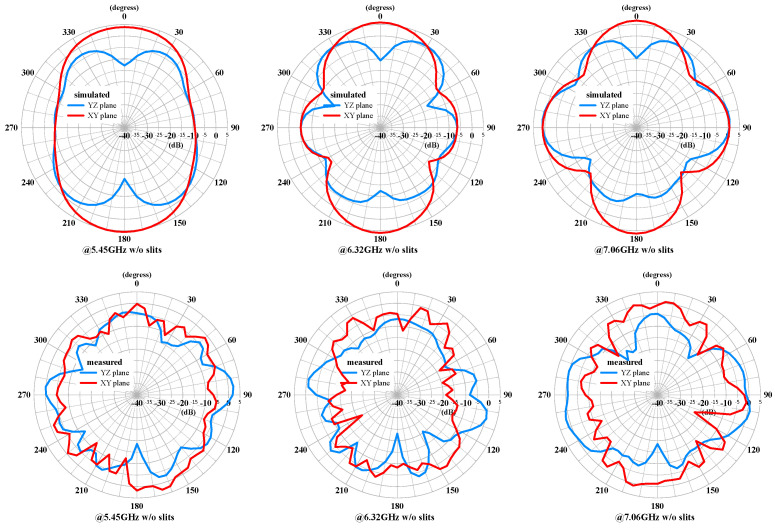
The measured and simulated radiation patterns of the MIMO antenna with slits at 5.45 GHz, 6.32 GHz, and 7.06 GHz.

The measured and simulated radiation patterns of the MIMO antenna with both antennas excited are shown in Figure 18 and Figure 19, respectively. The patterns demonstrate good directional coverage in the azimuth angle (XY plane). The gain decreases noticeably as the frequency increases. The elevation angle (YZ plane) also exhibits a similar trend and is not smooth. Due to the measured frequency points not resonating at the same frequencies as the simulated points, there are notable differences, especially at 4.41 GHz, where it is not even below −6 dB. 

### 3.3. Envelope Correlation Coefficient (ECC)

**Figure 20:**
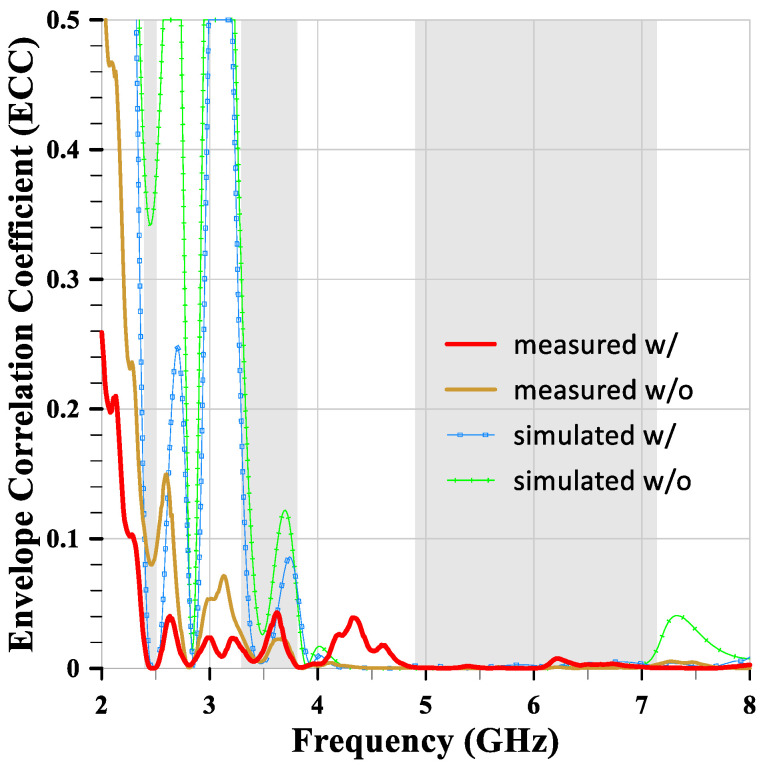
The measured and simulated ECC values of the MIMO antennas with and without slits.

The Envelope Correlation Coefficient (ECC) is a crucial parameter for evaluating MIMO antenna systems. It quantifies the correlation between the signals received or transmitted by different antenna elements in a MIMO system. ECC values range from 0 to 1, where lower values indicate better performance. Typically, an ECC value below 0.5 is considered acceptable for MIMO antenna systems. This threshold indicates that the antenna elements are sufficiently independent, effectively providing spatial diversity, which leads to improved performance in terms of data rate and reliability. In practice, ECC is often approximated using S-parameters (3). Figure 20 shows the ECC value of the MIMO antenna with slits is less than 0.04 within all required bands. Obviously, the antenna elements are sufficiently independent, providing spatial diversity, high data rates, and reliability.

(3) $$ECC\approx \frac{{\left|S_{11}^{*}S_{12}+S_{21}^{*}S_{22}\right|}^{2}}{\left(1-{\left|S_{11}\right|}^{2}-{\left|S_{21}\right|}^{2}\right)\left(1-{\left|S_{22}\right|}^{2}-{\left|S_{12}\right|}^{2}\right)}\;$$

### 3.4. Channel Capacity Loss (CCL)

The Channel Capacity Loss (CCL) is also an important parameter for evaluating MIMO antenna systems. The CCL value represents the data loss in bits per second per Hertz due to channel conditions. Therefore, the smaller the CCL value, the better. A CCL value below 0.4 bits/s/Hz can be considered excellent channel conditions. Formulas (4)–(7) are used to calculate the CCL values.

(4) $${CCL=-log}_{2}det\;{(\psi }^{R})$$

where $\psi^{R}$ is the correlation matrix at the receiving antenna.

(5) $$\psi^{R}=\left[\begin{array}{cc} \rho_{11} & \rho_{12}\\ \rho_{21} & \rho_{22} \end{array}\right]$$

(6) $$\rho_{ii}=1-{\left|S_{ii}\right|}^{2}-{\left|S_{ij}\right|}^{2},\;for\;i,\;j=1\;or\;2$$

(7) $$\rho_{ij}=-\left(S_{ii}^{*}S_{ij}+S_{ji}^{*}S_{jj}\right),\;for\;i,\;j=1\;or\;2$$

**Figure 21:**
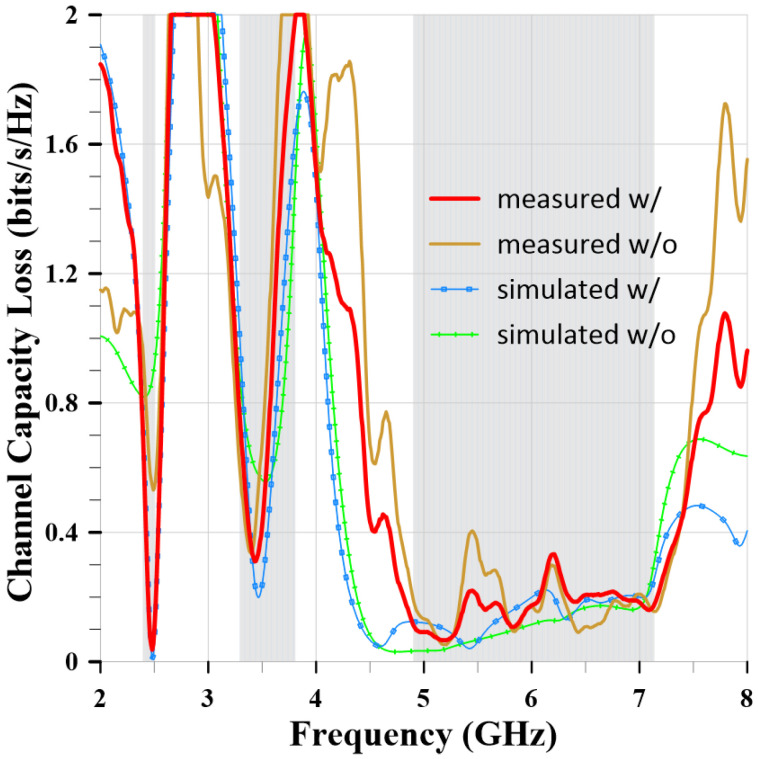
The measured and simulated CCL values of the MIMO antennas with and without slits.

Figure 21 shows the measured and simulated CCL values of the MIMO antennas with and without slits. The red curve represents the measured CCL of the MIMO antenna with slits. The results represent CCL values below 0.4 bits/second/Hz in the desired frequency band. This means that when the MIMO antenna is used in high data rate communication environments, fewer data bits will be lost. 

## 4. Comparative Analysis

Table 2 shows the comparison of the proposed MIMO antenna with other designs. The proposed MIMO antenna’s decoupling method effectively suppresses mutual coupling at two frequencies, which can be designed to the required frequencies. This design does not require additional components and does not increase the overall dimensions of the MIMO antenna. From Table 2, it is evident that dipole or monopole antennas cannot achieve the required designable suppression frequencies; only patch antennas can. However, those patch antennas are designed for only one frequency. Therefore, only the proposed MIMO antenna can satisfy the design requirements for multifrequency coupling suppression with a monopole antenna type. 

## 5. Conclusions

In this paper, the authors discuss the evolutionary steps of the compact multiband monopole antenna and its current distribution, which result in impressive wide bandwidth, radiation efficiency, radiation patterns, and gains. The overall performance of the compact multiband monopole antenna makes it highly suitable for compact devices such as IoT sensors. Furthermore, the authors also discuss a method to suppress the mutual coupling between two closely spaced antennas. The method is simple to configure, requires no additional components, and achieves excellent isolation. It will significantly increase the SNR and data throughput. This makes it highly suitable for MIMO antenna systems, such as WiFi-7 infrastructure. The measurement results are in good agreement with the simulations, validating the performance of the compact multiband monopole antenna and the mutual coupling suppression method for the MIMO antenna. Furthermore, these antennas are suitable for a variety of compact sensor devices and wireless infrastructure applications.

## Figures and Tables

**Table 1:** The dimensions of the compact multiband monopole antenna.

$L_{m}$	$W_{m}$	$L_{a}$	$L_{a1}$	$L_{a2}$	$L_{a3}$	$L_{a4}$	$W_{a}$	unit
30	10	5	14.5	15	2	11	3	
$W_{a1}$	$W_{a2}$	$W_{a3}$	$L_{s}$	$W_{s}$	$L_{g}$	$W_{g}$	h	mm
0.5	1	3.5	6	0.6	4.5	4	1.6	

**Table 2:** Comparison of the proposed MIMO antenna with other designs.

Ref.	$\mathrm{Size}\;({mm}^{3})$	Decoupling Method	Isolation (dB)	ECC	Designable Suppression Frequencies (GHZ)	Add Components	No. of Antennas
This paper	30 × 40 × 1.6	DGS	−26, −13	<0.04	2.46, 3.47	x	2 monopoles
[21]	x	Hilbert-shaped WG-MTM	−21.4	x	3.5	x	2 patches
[20]	x	CSRs WG-MTM	−22.86	x	3.5	x	2 patches
[19]	40 × 58.3 × 4.75	H-RRs MTM	−30	<0.14	5.9	v	2 patches
[18]	66 × 66 × 0.13	Pol. direction	<−20	<0.005	x	x	4 dipoles
[17]	41 × 41 × 1.6	Pol. direction and DGS	−21	0.032	x	x	4 monopoles
[13]	90 × 40 × 0.79	neutralization lines	<−17	<0.005	x	x	2 monopoles
[12]	36 × 18 × 1.6	T-shaped stub	<−18	<0.006	x	x	2 monopoles
[8]	x	Insulator	−29	x	5	x	2 patches

## Data Availability

The data presented in this study are available via email upon request to the corresponding author.

## References

[1] Khorov E., Levitsky I., Akyildiz I.F. (2020). Current Status and Directions of IEEE 802.11be, the Future Wi-Fi 7. IEEE Access.

[2] 5G-Americas Advanced Antenna Systems for 5G White Paper. https://www.5gamericas.org/advanced-antenna-systems-for-5g/.

[3] Farasat M., Thalakotuna D.N., Hu Z., Yang Y. (2021). A Review on 5G Sub-6 GHz Base Station Antenna Design Challenges. Electronics.

[4] Kolangiammal S., Balaji L., Mahdal M. (2023). A Compact Planar Monopole UWB MIMO Antenna for Short-Range Indoor Applications. Sensors.

[5] Kempanna S.B., Biradar R.C., Ali T., Jhunjhunwala V.K., Soman S., Pathan S. (2023). A Compact Slotted UWB Antenna Based on Characteristics Mode Theory for Wireless Applications. Designs.

[6] Zhu J., Eleftheriades G.V. (2010). A Simple Approach for Reducing Mutual Coupling in Two Closely Spaced Metamaterial-Inspired Monopole Antennas. IEEE Antennas Wirel. Propag. Lett..

[7] Haskou A., Pesin A., Le Naour J.Y., Louzir A. Compact, Two-Port, Slot, Antenna for Dual-Band WiFi 2 × 2 MIMO Applications. Proceedings of the 2019 49th European Microwave Conference (EuMC).

[8] Muhsin M.A., Rahim S.A., Nor M.M. Dual-Element MIMO Antennas with Rectangular Strip Line Insulator for Wi-Fi hotspots Application. Proceedings of the 2019 IEEE Asia-Pacific Conference on Applied Electromagnetics (APACE).

[9] Bukhari B., Rather G.M. Multiband Compact MIMO Antenna for sub-6 GHz Applications. Proceedings of the 2022 IEEE Microwaves, Antennas, and Propagation Conference (MAPCON).

[10] Srinivas G., Jabin D., Singh A.K. Multiband MIMO Antenna with Reduction in Mutual Coupling and ECC. Proceedings of the 2014 Students Conference on Engineering and Systems.

[11] Shiddanagouda F.B., Kumar N.D., Vani R.M., Hunagund P.V. Two Element Multiband MIMO Antenna for WiFi/5G/WLAN Band Applications. Proceedings of the 2021 IEEE Microwave Theory and Techniques in Wireless Communications (MTTW).

[12] Aung M.S., Hla T.T. Two-Port Wideband MIMO Antenna for Sub-6GHz 5G Applications. Proceedings of the 2024 IEEE Conference on Computer Applications (ICCA).

[13] See C.H., Abd-Alhameed R.A., Abidin Z.Z., McEwan N.J., Excell P.S. (2012). Wideband Printed MIMO/Diversity Monopole Antenna for WiFi/WiMAX Applications. IEEE Trans. Antennas Propag..

[14] Xu H.-X., Wang G.-M., Lv Y.-Y., Qi M.-Q., Gao X., Ge S. (2013). Multifrequency monopole antennas by loading metamaterial transmission lines with dual-shunt branch circuit. Prog. Electromagn. Res..

[15] Xu H.-X., Wang G.-M., Qi M.-Q., Zhang C.-X., Liang J.-G., Gong J.-Q., Zhou Y.-C. (2013). Analysis and Design of Two-Dimensional Resonant-Type Composite Right/Left-Handed Transmission Lines With Compact Gain-Enhanced Resonant Antennas. IEEE Trans. Antennas Propag..

[16] Das N., Islam M.T., Mia M.S., Moniruzzaman M., Mostafa A., Azim R. A Four-element MIMO Antenna for WiFi, WiMAX, WLAN, 4G, and 5G sub-6 GHz Applications. Proceedings of the 2022 12th International Conference on Electrical and Computer Engineering (ICECE).

[17] Das N., Mia M.S., Islam M.T., Mostafa A., Dhar K., Azim R. A Dual-band MIMO Antenna for 5G sub-6 GHz/WiFi/WiMAX/WLAN/Bluetooth/C-band Applications. Proceedings of the 2023 International Conference on Electrical, Computer and Communication Engineering (ECCE).

[18] Abdelghany M.A., Ibrahim A.A., Mohamed H.A., Tammam E. (2024). Compact Sub-6 GHz Four-Element Flexible Antenna for 5G Applications. Electronics.

[19] Khan M.S., Khan S., Khan O., Aqeel S., Gohar N., Dalarsson M. (2023). Mutual Coupling Reduction in MIMO DRA through Metamaterials. Sensors.

[20] Xu H.X., Wang G.M., Qi M.Q., Zeng H.Y. (2012). Ultra-small single-negative electric metamaterials for electromagnetic coupling reduction of microstrip antenna array. Opt. Express.

[21] Xu H.-X., Wang G.-M., Qi M.-Q. (2013). Hilbert-Shaped Magnetic Waveguided Metamaterials for Electromagnetic Coupling Reduction of Microstrip Antenna Array. IEEE Trans. Magn..

[22] Lin C.-K., Lin D.-B., Yu C.-K. A Compact Multiband Monopole Antenna for Wi-Fi Applications. Proceedings of the 2024 IEEE International Workshop on Antenna Technology (iWAT).

